# Supplementary figures and images for: Comprehensive characterization of plasma cell-free Echinococcus spp. DNA in echinococcosis patients using ultra-high-throughput sequencing
Source: PLoS Negl Trop Dis. 2020 Apr 13;14(4):e0008148. doi: 10.1371/journal.pntd.0008148 (PMC7209354; doi:10.1371/journal.pntd.0008148)

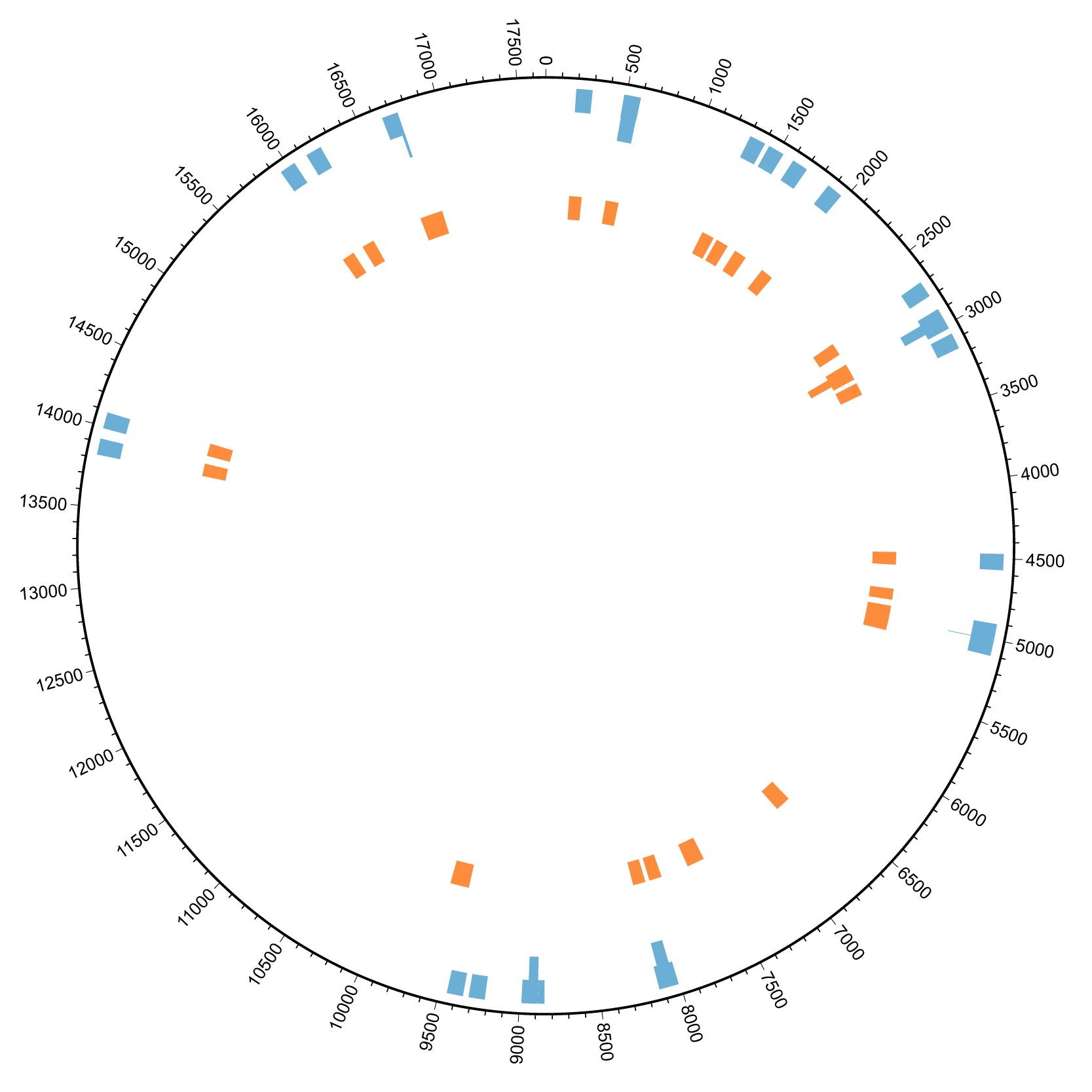

Supplement: S1 Fig — The circulation genome visualization showed the E. granulosus reads mapping position (outermost blue circle). The inner orange circle represents the count of patients with reads detected in the region. (TIFF) [file pntd.0008148.s006.tiff]

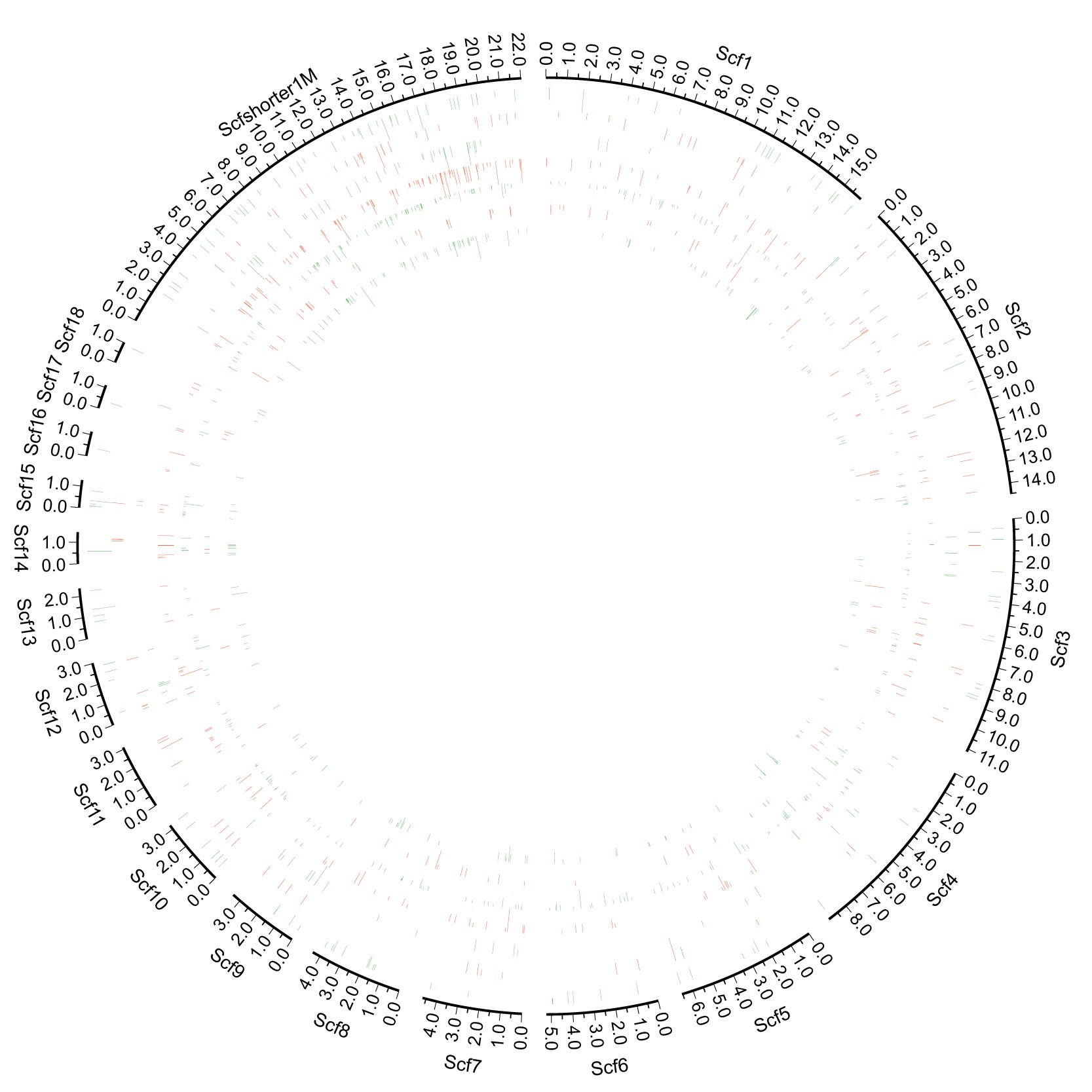

Supplement: S2 Fig — Seven E. granulosus samples detected with more than 100 Echinococcus spp. read pairs were displayed based on the nuclear genome. Green and red circles indicate different samples. (TIF) [file pntd.0008148.s007.tif]

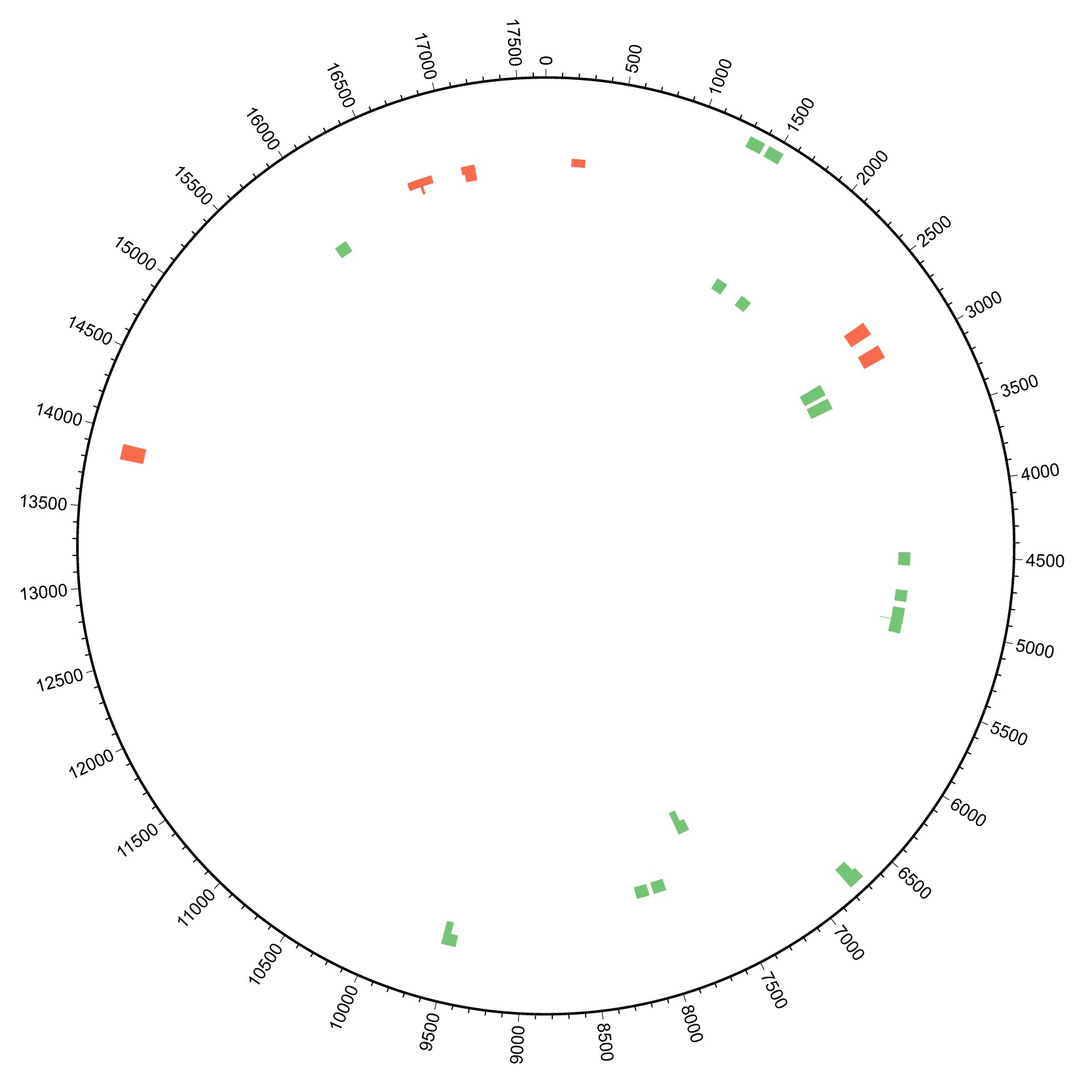

Supplement: S3 Fig — Seven E. granulosus samples detected with more than 100 Echinococcus spp. read pairs were displayed based on the mitochondrial genome. Green and red circles indicate different samples. (TIF) [file pntd.0008148.s008.tif]

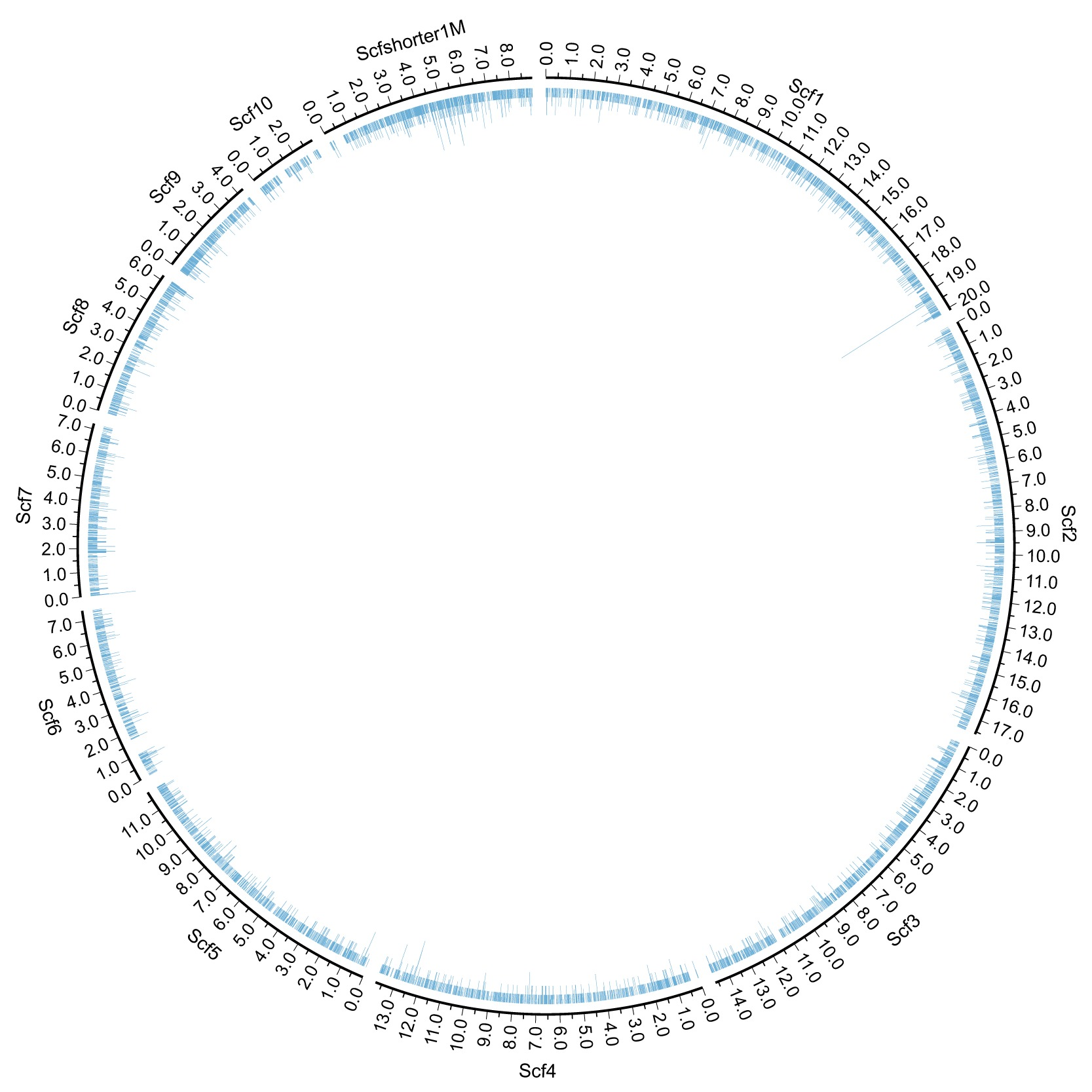

Supplement: S4 Fig — The circulation genome visualization showed the E. multilocularis reads mapping position (outermost blue circle). Ten scaffolds longer than 1Mb were displayed in the separate fragment (Scf1-Scf10). Scaffolds shorter than 1Mb were concatenated to display (Scfshort1M). (TIF) [file pntd.0008148.s009.tif]

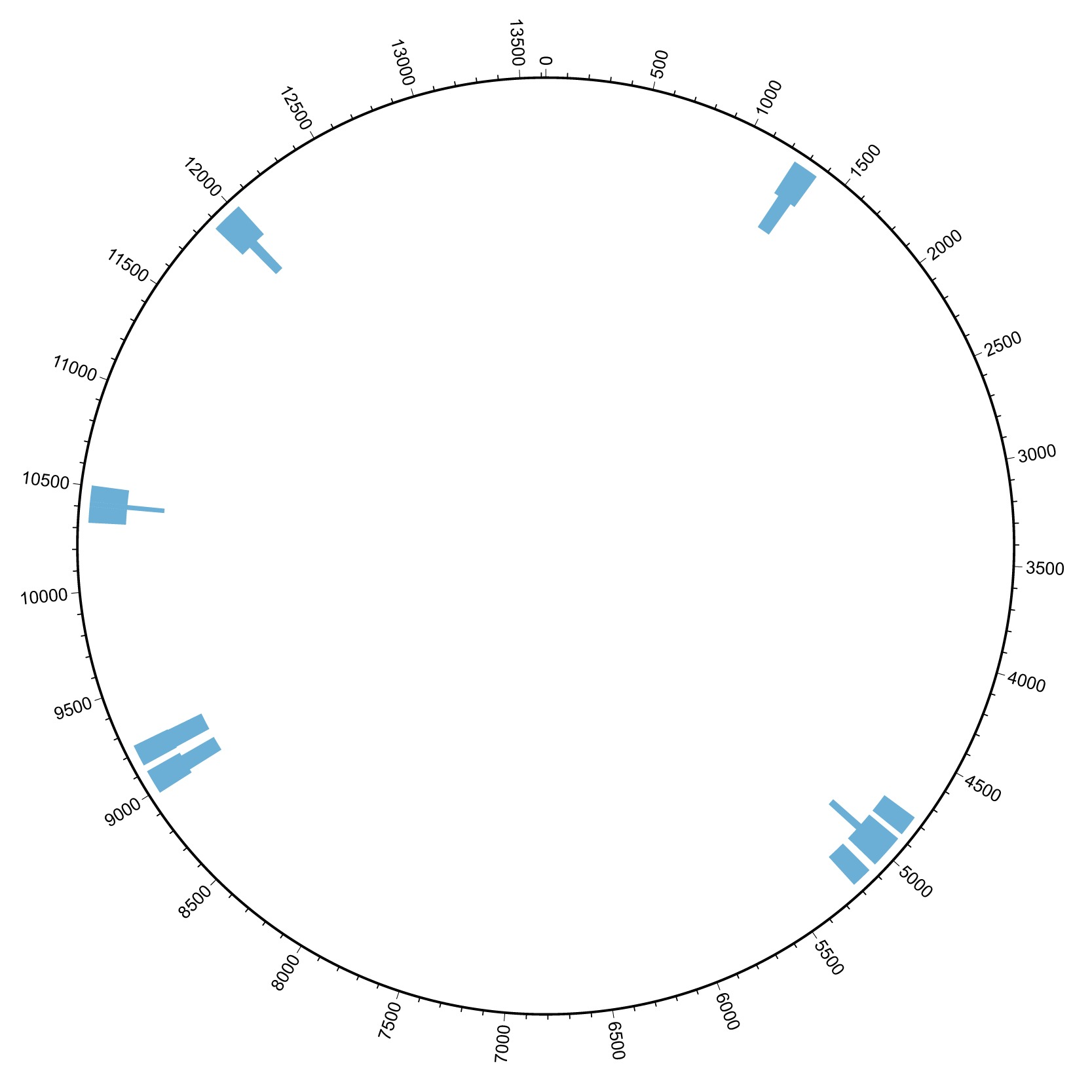

Supplement: S5 Fig — The circulation genome visualization showed the E. multilocularis reads mapping position (outermost blue circle). (TIF) [file pntd.0008148.s010.tif]

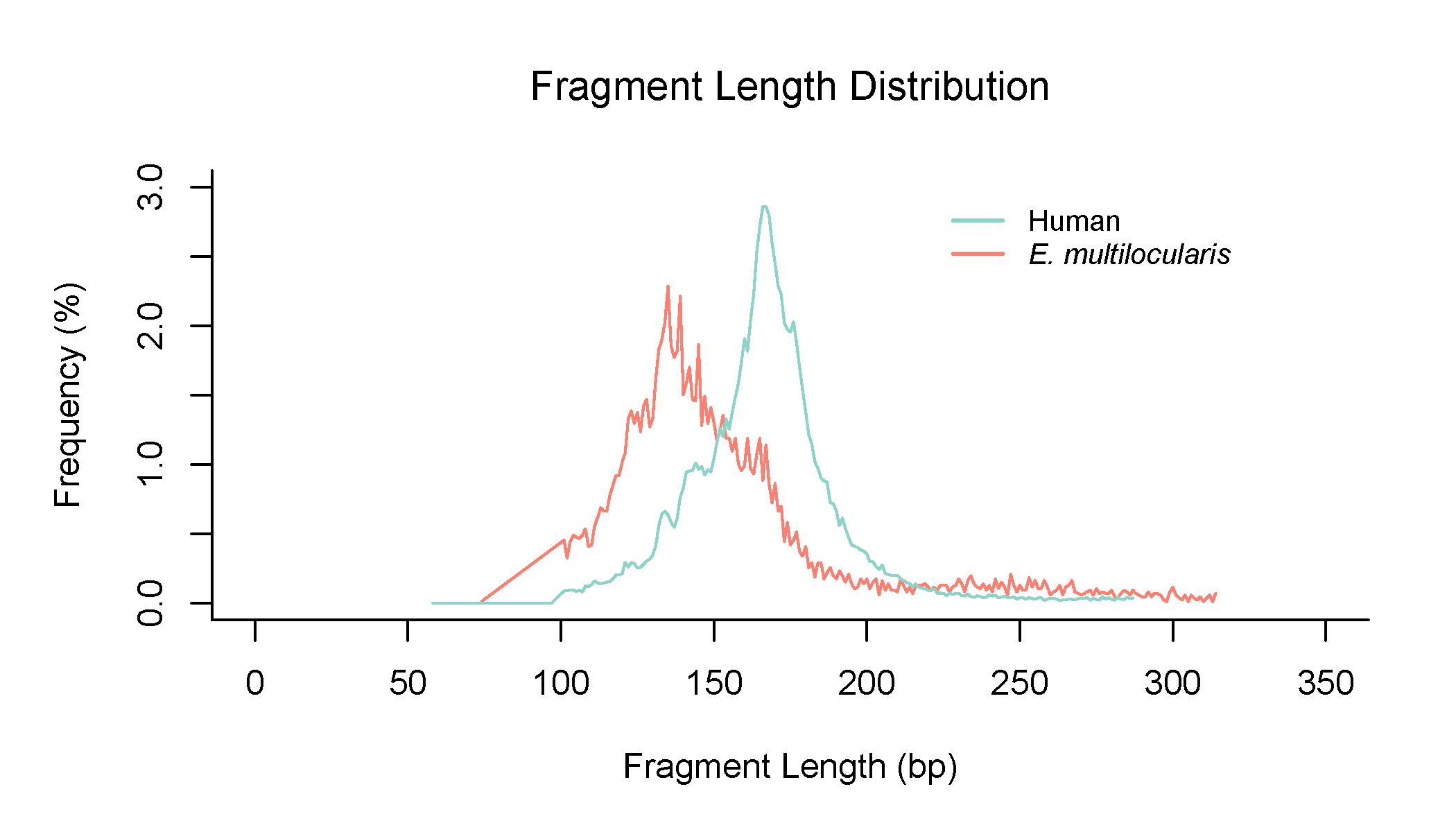

Supplement: S6 Fig — Overall fragment length distribution of E. multilocularis cfDNA was shorter than that of humans. (TIFF) [file pntd.0008148.s011.tiff]
