# Supplementary material for: Comprehensive characterization of plasma cell-free Echinococcus spp. DNA in echinococcosis patients using ultra-high-throughput sequencing
Source: PLoS Negl Trop Dis. 2020 Apr 13;14(4):e0008148. doi: 10.1371/journal.pntd.0008148 (PMC7209354; doi:10.1371/journal.pntd.0008148)
Supplement: Attachment — Submitted filename: Letter_to_editor_and_Reviewers.docx [file pntd.0008148.s012.docx]

Dear Editors,

(Guest Editor: Uriel Koziol; Deputy Editor: Adriano Casulli)

Thank you for the time and effort in reviewing our manuscript entitled "Comprehensive characterization of plasma cell-free *Echinococcus* spp. DNA in echinococcosis patients using extremely high throughput sequencing" (#PNTD-D-19-01705) to *PLOS Neglected Tropical Diseases*. We appreciate you and each of the reviewers’ feedback and revised our manuscript accordingly.

Attached please find our letter with the response to the reviewers.

**Editor’s general comment:**

All reviewers were generally positive regarding the manuscript and have stated that it represents a valuable contribution to the field. However, all of the reviewers expressed concerns regarding specific aspects of the manuscript and the experimental work. In particular, one main issue that two reviewers found was that only one case of *E. multilocularis* was investigated, and recommend either incorporating additional cases or removing this case from the manuscript (and from the title). In any case, this aspect needs to be addressed appropriately in the discussion. All reviewers also consider that the discussion should include a guideline on how cfDNA could (or could not) be used clinically in the near future as a method for diagnostics. Additional concerns should also be addressed, particularly regarding the negative controls, which should be described and presented in far greater detail. Finally, make sure to include the permit number for ethical approval of this study in the ethics statement, and to make the original data for the negative controls available as well.

**Response:**

Thanks for your kind feedback. Please find our response to the reviewers’ comments one by one below (including our response to the Editor’s general comments).

We echo the concerns of the reviewers regarding the issue of the limited sample of *E. multilocularis*. Fortunately for local residents, but unfortunately for us as researchers, the circulating *Echinococcus* spp. in Tibet is mainly *E. granulosus*. According to *Echinococcosis in China, a Review of the Epidemiology of Echinococcus spp.* [1], there are three foci of *E. multilocularis* distribution in China, including a tiny tip of northeast Tibet, bordering Sichuan province. We have cited the map from this review for your kind reference. Figure taken from (1), the three foci of *E. multilocularis* in China were circled areas (I, II, and III). The red arrow pointed towards the *E. multilocularis* focus in Tibet (F), bordering Sichuan Province (E). As a result, AE patients from Tibet mainly go to Sichuan for medical treatment. Consequently, we did not identify enough AE patients. Future research can focus on *E. multilocularis* and collect samples in the three foci in China, and prove if our finding with *E. multilocularis* is true.

For the special characteristics (overall shorter length and higher concentration) of cell-free *E. multilocularis* DNA in plasma, and for only one accidental case sample we collected, we have downplayed the description and made it more as a hypothesis to be tested in future studies. What is more, our hypothesis is reasonable. The metacestodes of *E. multilocularis* and *E. granulosus* have different morphology. *E. granulosus* has a unique cyst formation and the cyst is usually clearly separated from the surrounding host tissues. Conversely, the *E. multilocularis* metacestode has an infiltrating lesion, which looks more like a malignant tumor, continuously damaging the liver or other target organs. As a matter of fact, a nickname of *E. multilocularis* in Chinese is “Chong Ai”, or parasite cancer as the literal translation. Our finding is in support of this hypothesis that the aggressive metacestode of *E. multilocularis* may have more cfDNA released to host circulation like a malignant tumor and it acted as so. This is in line with the previous finding that the sensitivity of PCR-based methods in AE samples was higher than in CE samples [2–4]. Having said that, this phenomenon needs more research to validate, and we hope our report can give some inspiration to other researchers with more *E. multilocularis* samples.

We agree with the editor and reviewers’ point that the discussion should include a guideline on how cfDNA could (or could not) be used clinically in the near future as a method for diagnostics. As for the clinical diagnostic usage of cfDNA, we think the sequencing of cfDNA was not suitable for routine clinical examination yet, but it can be used for differential diagnosis, in which existing clinically methods cannot give clear conclusions. For example, the CL (Cystic Lesion) patients can be further diagnosed with plasma cfDNA sequencing and avoid the risk of invasive diagnosis. The high cost is an important factor hindering its application. Although the cost of sequencing continues to decrease, due to the low concentration of cell-free *Echinococcosis* spp. DNA in plasma, a lot of sequencing data is still needed. The even distribution of cell-free *Echinococcus* spp. reads on genome makes PCR unsuitable for their detection. To increase the application of cfDNA, we could think of some areas to be explored in future studies, for example, capturing cell-free *Echinococcus* spp. DNA with probes covered the whole genome and enriching the concentration of cell-free *Echinococcus* spp. DNA by host sequence removal.

We also totally agree with your suggestion that our negative controls should be described and presented in far greater detail. We have added the detailed description in the methods section “2.8 Workflow evaluation” Lines 298-306. To evaluate the reliability of the workflow, we used three datasets, including simulated data, cell lines deep sequencing data, and cfDNA sequencing data of individuals from non-endemic areas. Simulated data (Paired-end 100bp) were produced by wgsim (<https://github.com/lh3/wgsim>) with human reference and *Echinococcus* spp. sequences. Data of cell lines produced with the same sequencing platform were used as a negative control. CfDNA sequencing data of 107 pregnant women from an ongoing study from non-endemic areas were also used as a negative control. All three datasets were analyzed with the workflow to evaluate its performance. The evaluation of three datasets all verified the reliability of our workflow.

We have made the simulated data and cell lines sequencing data available at CNGB (CNP0000857). The cfDNA sequencing data of the pregnant women are from ongoing research, only partial data of these 107 individuals can be made available. That is, the candidate *Echinococcus* spp. reads (produced from Kraken step, Fig 1) after removing human sequences of these 107 individuals are made available at CNGB (CNP0000868).

We thank the reviewer for the kind reminder on the inclusion of the permit numbers for ethical approval of this study in the ethics statement. Permit numbers for the ethical approval of this study have been included in the “2.1 Ethics Statement” Lines 201-204.

**Reviewers’ Comments**

**Reviewer 1 Comments:**

Ji et al. report on work concerning the detection of cell-free parasite DNA in serum samples of echinococcosis patients by Next Generation Sequencing approaches. The authors identified parasite DNA in 23 samples (of 23 different patients). They demonstrated that parasite cfDNA concentrations are very low in echinococcosis patients, which could explain why several PCR approaches towards this aim so far failed (or only showed very low sensitivity). To my knowledge, successful detection of cfDNA in echinococcosis patients by high throughput NGS has so far not been published which is, indeed, a strong point of this work. I agree with the authors that basic knowledge on cfDNA realeased by these parasites into host serum is important for future development of respective molecular diagnostics methodology (although, of course, not feasible at the moment due to high costs). In general, the work is well written and the NGS methods seem to have been carried out in a professional way (although a bioinformatics expert should closer evaluate this part of the work). There are, however, also several limitations in this work. On the one hand, the authors do not provide any suggestions on how, based on their data, proper molecular diagnostics of serum samples can be carried out in the future (their data rather suggest that this will not be possible for long time – which, of course, would be a valid result if properly discussed). The most serious limitation I see is that only one *E. multilocularis* sample was analyzed. This is surely not enough to come to significant conclusions, particularly concerning the general suitability of echinococcosis serum samples for molecular diagnostics (in *E. multilocularis* the cfDNA concentration was much higher), or concerning differential diagnostic methods that can distinguish between *E. granulosus* and *E. multilcularis*. On the one hand, this could be solved by taking out the *E. multilocularis* sample and by concentrating only on *E. granulosus* (stating, in the end, that it is almost impossible to detect *E. granulosus* DNA in plasma samples). On the other hand, I would consider a significant step forward (i.e. ‚importance‘ as outlined in the PNTD guidelines for publication) in echinococcosis diagnostics the comprehensive analysis of both *E. multilocularis* and *E. granulosus* cfDNA in patient plasma. I thus strongly suggest that additional *E. multilocularis* samples are analyzed so that the title of the study (Comprehensive characterization of cf Echinococcus spp. DNA….) is justified.

**Reply:**

We thank the reviewer for your kind advice.

We agree with the reviewers’ point that the suggestion and discussion should include a guideline on how cfDNA could be used clinically in the near future as a method for diagnostics. As for the clinical diagnostic usage of cfDNA, we think the sequencing of cfDNA was not suitable for routine clinical examination yet, but it can be used for differential diagnosis, in which existing clinically methods cannot give clear conclusions. For example, the CL (Cystic Lesion) patients can be further diagnosed with plasma cfDNA sequencing and avoid the risk of invasive diagnosis. The high cost is an important factor hindering its application. Although the cost of sequencing continues to decrease, due to the low concentration of cell-free *Echinococcosis* spp. DNA in plasma, a lot of sequencing data is still needed. And evenly distribution of cell-free *Echinococcus* spp. reads on genome makes PCR unsuitable for their detection. To increase the clinical availability of cfDNA, we could think of some areas to be explored in the future study, for example, capturing cell-free *Echinococcus* spp. DNA with probes which could cover the whole genome, and enriching the concentration of cell-free *Echinococcus* spp. DNA by host sequence removal. And we have added discussion in Lines 626-636.

As for the *E. multilocularis* sample, we share the concern of the reviewer. For the features of shorter length and a higher concentration of cell-free *E. multilocularis* DNA in plasma, and for only one accidental case sample we collected, we have downplayed the description in the manuscript and made it more as a hypothesis, which is to be tested in future studies. What is more, our hypothesis is reasonable. The metacestodes of *E. multilocularis* and *E. granulosus* have different morphology. *E. granulosus* has a unique cyst formation and the cyst is usually clearly separated from the surrounding host tissues. Our finding is in line with this hypothesis that the aggressive metacestode of *E. multilocularis* may have more cfDNA released to host circulation like a malignant tumor and it acted as so. Having said that, this phenomenon needs more research to validate, and we hope our findings can give some inspiration to other researchers.

Additional points

The English of the manuscript is generally quite well and comprehensible.

1) Nevertheless, another round of checking readability would be good (particularly proper use of specifying articles like ‚the‘ and ‚a‘ as well as correct use of plural/singular).

**Reply:** Thank you for your careful review. The lexical errors have been checked and corrected.

2) L146-148: why is it likely that parasite DNA can be found in plasma because of complex host-parasite interaction? This kind of interaction could exclusively be mediated by proteins.

**Reply:** We apologize for the confusion. Our description here is not accurate enough, and we have corrected the description from Line 157 to 160. The parasite can survive in the host for a long period of time by the secretion of proteins and regulation of host immune response. As for parasite cfDNA, the metabolic activities of the parasites and attacks from the host’s immune system may cause the parasite’s DNA to be released into the host's circulatory system, and the possible mechanisms can be summarized as active secretion and passive release [5].

3) Shouldn’t there be a permit no. for the ethics statement?

**Reply:** It was added in the manuscript as in Lines 201-204.

4) Paragraph 2.2.: please be specific on when the plasma samples have been taken. I guess it was before (!) treatment. This should be pointed out in detail.

**Reply:** All the plasma samples were taken before treatment, and we have described this in more detail in the revised manuscript in Line 210.

5) Paragraph 2.5.: please be specific on when the ELISA has been carried out.

**Reply:** ELISA was carried out before treatment, and we have pointed out this in the revised manuscript in Line 254.

6) Related to point 4 and 5: it would, of course, strengthen the study if cfDNA analyses (and maybe ELISA) would have been carried out also after treatment (especially chemotherapy) at least for some samples.

**Reply:** Thanks for your suggestion. The surveillance of post-intervention is also an important topic. The current methods of patient monitoring rely mainly on imaging and serology, which provide limited information on treatment effects. CfDNA as a direct biomarker released by the parasite may have potential usage in treatment monitoring, and this part can be considered as an extension of this study in the future.

7) The authors only superficially discussed why they detected so much more cfDNA in the *E. multilocularis* patient. I think the big difference is that *E. multilocularis* metacestode material in the human host is generally a mixture between nectrotic parasite tissue and actively proliferating tissue. Hence, cfDNA could result from parasite cell necrosis. This should be taken into account and could also explain why there were shorter fragments in the *E. multilocularis* sample.

**Reply:** We thank the reviewer for the kind input. Indeed, compared with *E. granulosus*, the metacestode of *E. multilocularis* is an infiltrating lesion, and high concentration and short fragments of the parasite in the *E. multilocularis* sample could be due to the mixture of necrotic parasite tissue and actively proliferating tissue. We have added a discussion about this from Line 504 to 515 and Line 596 to 602.

8) L 437/438: shouldn’t it be possible to calculate reads per genome size? This would give us hints as to whether nuclear or mitochondrial genome are over-represented.

**Reply:** Thanks for your suggestion, and we have calculated this value (S2 Table) and described the results from Line 428 to 436. The results showed that although most reads are derived from the nuclear genome, the mitochondrial genome has more reads detected per genome size, which may be because of the multiple copies of mitochondria [6]. The discussion about this is in Lines 570-571.

9) Fig. 3: At least in the mitochondrial genome I see some hotspots. Which are these?

**Reply:** We thank the reviewer for the sharp observation and have checked the mitochondrial data accordingly. The region appears to be the tandem repeat region which was poorly assembled. We have therefore updated the figure with the latest *E. granulosus* mitochondrial reference genome with a longer sequence [7], and afterward, the original hotspots look more even in the updated figure (S1 Figure). Based on the long reads sequencing, Kinkar et al., have assembled complete mitochondrial genome (17,675bp), which was about 4.4 Kb longer than the existing one (13,599bp), and the 4.4 Kb newly assembled part belonged to the tandem repeat region. Repeat regions are the most difficult part of genome assembly, and the repeat regions of the most existing genome were not well assembled including *Echinococcus* spp. genomes. Reads from these repeat regions aligned to the poorly assembled genome may seem like some hotspots. We have also added the discussion about reference genome quality and cfDNA distribution in Lines 576-581.

10) Table 1: please add for all samples which species was identified according to NGS reads.

**Reply:** We have added this to Table 1. The species identified with NGS reads are consistent with PCR of lesion tissues. For the short length of reads, the similarity of different *Echinococcus* species and limited reference genomic sequences, some reads can’t be classified to their corresponding species, but most reads of the sample are classified to the species they belong to. The detailed classified results of the read counts are presented in S3 Table.

11) References: genus/species in italics.

**Reply:** We thank the reviewer for the careful observation. Genus/species in references have all been corrected to italics.

**Reviewer 2 Comments:**

Methods: The objectives are clearly stated. The study design is appropriate but there are some negative control results that should be presented and discussed (these dataset is mention but missing in the text). The sample size is ok. No concerns about ethical or regulatory requirements.

**Reply:** We thank the reviewer for your kind suggestion. we have added a description of the negative control sample in the methods section “2.8 Workflow evaluation” Lines 303-306. We have summarized the detailed results describing the control dataset into section “3.2 Performance evaluation of the analysis workflow” Lines 370-374.

**Results:** Some negative control results are mention but need to be presented and discussed. The figures 3 and 4 need to be improved. Some statistical analysis/simulations in relation to the number of reads generated (more than 80 millions) and the number of reads detected may give some more support to presented results.

**Reply:** We thank the reviewer for this comment.

We have summarized the detailed results describing the control datasets in section “3.2 Performance evaluation of the analysis workflow” in Lines 370-374. No *Echinococcus* spp. reads were detected from negative control data. To evaluate the workflow, we also generated simulated reads from human and *Echinococcus* spp. references (including 300,000,000 paired-end reads from humans, 1,000 paired-end reads from *Echinococcus* spp. nuclear genome, and 100 paired-end reads from *Echinococcus* spp. mitochondrial genome) and analyzed these data with our workflow. After analysis with the workflow, 99.5% of the *Echinococcus* spp. nuclear genome reads were identified, 98.0% of the *Echinococcus* spp. mitochondrial genome reads were identified, and no human reads were wrongly identified (S1 Table).

Figures 3 and 4 have been improved. To get higher resolution figures and clearly display the distribution of *Echinococcus* spp. reads on the genome, the figures were divided into single Circos figure to display, and the representative Circos figure of *E. granulosus* nuclear genome (Fig 3) was placed in the manuscript and other figures were supplementary materials. The inner-circle about genome annotation that was not particularly informative was replaced with information on patient counts.

To analyze the relationship between the number of reads generated and the number of reads detected, we have added statistical analysis in the section “2.10 Analysis of sequencing data volume and positive detection” from Line 330 to 347. Detailed reply about this question was also described below at the “Major comments Point 2”.

**Conclusions:** The identification of *Echinoccoccus* spp. cell free DNA reads is really marginal in some patients, however the authors conclude that the technique es valid in 100% cases. This issue should be discussed and mention in conclusions.

**Reply:** We thank the reviewer for this comment. We have added the discussion on this issue in Lines 532-539. The detection rate is mainly affected by the concentration of cell-free *Echinococcus* spp. DNA in plasma. The lower the concentration, the harder it is to be detected. Just like the cell-free DNA of fetal in maternal plasma, which are influenced by gestational age, maternal BMI, fetal aneuploidy status and other factors [8]. The concentration of cell-free *Echinococcus* spp. DNA might also be affected by many factors, such as disease status, parasite species, lesion size, and position, which need more comprehensive samples to explore its association with the different patterns.

**Summary and General Comments:**

Review of “Comprehensive characterization of cell-free *Echinococcus* spp. DNA in echinococcosis patients’ plasma using extremely high throughput sequencing” by Ji and co-authors.

In the present manuscript (MS) Ji and coauthors report the results of applying extremely high throughput sequencing to detecting cell-free *Echinococcus* spp. DNA in plasma of echinococcosis patients.
Authors do a well-organizer presentation of auspicious results regarding the possibility of identified and partially characterize *Echinococcus* spp. infections from human blood samples. The MS followed a straight-forward bioinformatic pipeline to identify reads from cell free DNA from *Echinococcus* and more importantly to discard false positive results. This approach has been successfully applied for others pathogens.

**Reply:** We thank the reviewer for the positive comment.

This MS is well written and my overall impression of it is positive, however I have some concerns that I think the authors need to address before publications. Some suggested changes could improve the robustness and presentation of the MS.
Major Comments:

1. Negative control data are not mention (Humans with no diagnostic *Echinoccoccus*) until the Results sections. This is a major problem since no results about this control dataset is presented in the MS (see Line 295, pg. 8). Given that detection of *Echinoccoccus* spp. reads is so marginal in the whole NGS dataset, the results of the bioinformatics pipeline applied to this 107 human blood samples are key to support presented positive results. Negative controls are not included under accession number of CNGB.

**Reply:** Thanks for your suggestion.

First of all, we have added a description of the negative control in the methods section under “2.8 Workflow evaluation” from Line 303 to 306. “Data of cell lines produced with the same sequencing platform were used as a negative control. CfDNA sequencing data of 107 pregnant women from an ongoing study living in non-endemic areas were also used as a negative control.”

After that, we have summarized the detailed results on the control dataset into section “3.2 Performance evaluation of the analysis workflow”. As for the negative controls, DNA of cell lines was sequenced and 2,047,723,953 paired-end clean reads were produced. Evaluation of the cell lines data with the workflow showed that no *Echinococcus* spp. reads were detected. Besides, control data from 107 individuals with a total of 6,838,155,312 paired-end clean reads were used to evaluate the workflow and *Echinococcus* spp. reads were not detected from these data.

We have made the cell lines sequencing data availability at CNGB (CNP0000857), which can be found in the “Data availability” section. As our study focuses on cell-free *Echinococcus* spp. DNA sequences identification and the cfDNA sequencing data of pregnant women is from ongoing research, only partial data of these 107 individuals can be made available. We have made the candidate *Echinococcus* spp. reads (produced from Kraken step) after removing human sequence of the 107 individuals available at CNGB (CNP000868).

2. The number of reads identified is rather marginal; this is particularly noticeable when considering the millions of reads generated in each sequencing experiment (median 235 million reads). Authors should discuss more thoroughly these results and possible improvements that need to be done in order to make this approach clinically feasible. For instance, I wonder if single reads could improve the detection of cell free DNA. In relation to this the authors should consider do some statistical/modeling analysis to estimate the minimal number of reads generated to get positive results.

**Reply:** We thank the reviewer for this suggestion.

We have added statistical analysis to estimate the minimal number of reads generated to get positive results in “2.10 Analysis of sequencing data volume and positive detection” from Line 330 to 347. The results were described from Line 413 to 420, and discussion on this could be found from Line 522 to 539. To estimate the minimal number of reads generated to get cell-free *Echinococcus* spp. DNA. detection, we treated sequencing as a random sampling process, and the number of sequencing reads as sampling times. By using hypergeometric distribution, we calculated the probability of each sample to get cell-free *Echinococcus* spp. DNA. detection with different sequencing volume (S4 Table). The detection of cell-free *Echinococcus* spp. DNA is mainly affected by two factors. One factor is the number of sequencing reads, and the other factor is the concentration of cell-free *Echinococcus* spp. DNA, which is the fundamental factor. The lower the concentration, the harder it is to detect, and concentrations can vary greatly from one individual to another. Just like the cell-free DNA of fetus in maternal plasma, which are influenced by gestational age, maternal BMI, fetal aneuploidy status and other factors [8]. The concentration of cell-free *Echinococcus* spp. DNA could also be affected by many factors, such as disease status, parasite species, lesion size, and position, which need more samples to explore.

**Minor Comments (not in order)**

- When building the *Echinococcus* spp. reference database, in order to reduce
  sequence contamination and get high quality genomes, authors used relatively high thresholds values (97% identity, 92% coverage, e-value) when filtering the possible sequences from host (human, mouse, etc..). Reads with high similarity to the host genomes are discarded. Authors need to clarify why these values are chosen, and as I understand the higher thresholds may allow including contaminant reads in the analysis.

**Reply:** We apologize for the confusion, and we have modified the description in this section “2.6 Database construction”. The reads mentioned in this part are pseudo-reads generated from *Echinococcus* spp. sequences. By pseudo-reads mapping, we defined the pseudo-reads with high similarity (identity ≥ 97%, coverage ≥ 92%, and e-value ≤ 1e-5) to host sequences as host contamination, and then we masked this host region on the *Echinococcus* spp. sequences (The process flow is in the left part of Fig 1). An identity of 97% is a widely-recognized threshold for species identification [9], and 92% coverage and the e-value could ensure the accuracy of the alignment. Indeed, as pointed out by the reviewer, this high threshold might leave some host contaminant sequences in the qualified *Echinococcus* spp. reference database, but it can also reduce wrongly removing of the *Echinococcus* spp. sequence. In the workflow of sequencing reads procession, we also chose the same high threshold to remove reads with poor mapping results (identity < 97%, coverage < 92%, and e-value > 1e-5) to *Echinococcus* spp. sequences (Fig 1, Step 5). Evaluation based on our simulated datasets and negative control dataset also verified the reliability of our parameters and workflow. The parameters can be further optimized when more reference genomes of host and *Echinococcus* spp. are available.

- I am not quite sure if Kraken is the best option to do the “mapping”. Please check or clarify.

**Reply:** We echo the concern of the reviewer and apologize for the confusion. We use Kraken just for initial screening, and there are multiple steps to filter the results after this step (Fig 1, steps 3, 4 and 5 in Lines 283-296). As most of the plasma cfDNA sequencing data belong to the host and Kraken can efficiently handle larger-scale data, we can reduce the amount of data to a small range with Kraken. In the following steps, we need to process this classified data and identify the high-quality mapping reads of *Echinococcus* spp. The pipeline has been tested with simulated data, and we could detect most of *Echinococcus* spp. reads without false positives.

- More analysis on the distribution of identified reads in the genome of E. spp. are needed. Are there any difference among patients? Which are the more represented regions? Is there any common characteristic in the overrepresented regions?

**Reply:** Thanks for your suggestions, and we have added the analysis of this part. The results were described from Line 450 to 454. “To analyze the overrepresented regions, we calculated the sample counts of the mapped regions. Most of the regions (211,342 bp, 98.95%) were covered with only one sample, less than 1% mapped length (1,983 bp, 0.93%) were covered with two samples, and very small region (262 bp, 0.12%) were covered with three samples”. The analysis results showed that most regions of the genome only with one sample covered. In order to compare the distribution of identified reads between different samples, we plot the coverage of the samples detected with more than 100 read pairs (S2 Fig and S3 Fig).

- Please check figures 3 and 4, both are not in resolution and are not particularly informative about genome distribution of reads.

**Reply:** Figures 3 and 4 have been improved. To get higher resolution figures and clearly display the distribution of *Echinococcus* spp. reads on the genome, the figures were divided into single Circos figure to display, and the representative Circos figure of *E. granulosus* nuclear genome (Fig 3) was placed in the manuscript and other figures are placed as supplementary materials (S1-S5 Figs). The less informative inner circles of genome annotation were removed, which was replaced by the patients' counts of the detected regions.

- Clarify the meaning of CL and CE in Table 1.

**Reply:** We thank the reviewer for the kind reminder. We have added footnotes accordingly in Table 1. CL (Cystic lesion) and CE (Cystic echinococcosis) correspond clinical-ultrasound examination typing of echinococcosis.

**Reviewer 3 Comments:**

**Methods**

The objectives of this paper were clearly described and study design is appropriate
As it could be considered as a pilot/exploratory study, number of included patient is correct.

**Reply:** We thank the reviewer for the positive comment.

My main concerns with this study are the following:

- among 23 patients, they had 22 CE and only one AE patient. They were included based on US examination. To my knowledge, US patterns of AE and CE are mostly different, except some cases (CL stage). In 22 out of their 23 cases, diagnosis of CE was known before biological diagnosis (molecular study). As diagnosis of type of echinococcosis is not the key point of results but a key point of methods, I suggest that this information should be given in methods and not in results section
**Reply:** We thank the reviewer for the kind suggestion. We highly appreciate the reviewer’s expert knowledge of AE and CE. Indeed, most of the ultrasound patterns of AE and CE are different, except in some CL stage. As a matter of fact, our only AE case at the beginning was diagnosed as CL stage with ultrasound examination, and the surgery lesion sample of this case was confirmed with PCR to be *E. multilocularis* infection. We have described this part in the methods accordingly, which could be found in section “2.2 Samples and processing” Lines 210-217.

- the authors used surprisingly two different strategies for DNA extraction : why ? which sample among the 23 was processed with alternative approach ? If they processed the AE blood sample with another DNA extraction approach than that used for CE blood sample, it affect significantly all the results obtained in this study. This information is not given in the paper.

**Reply:** We understand the concern of the reviewer and apologize for the confusion. There was only one sample (N4) from a CE patient with a larger volume of plasma available, thus it was processed with another kit. This was described in section “2.3 DNA extraction and high-throughput sequencing”, “Among the 23 plasma samples (S5 Table), 22 samples with volume 0.2 to 0.6 ml were extracted with MagPure Circulating DNA Mini KF Kit (Magen, Guangzhou, China), and one sample (N4) with volume 2.2 ml was extracted with QIAamp Circulating Nucleic Acid Kit (Qiagen, Hilden, Germany)”. The AE blood sample was processed with the same kit as the other 21 CE blood samples, thus the unique results of AE were not due to differences in lab procedures or testing kits. The detailed information on the kits we used is also shown in the S5 Table. The influence of the DNA extraction approach and other factors such as plasma volume to cell-free *Echinococcus* spp. DNA can be an interesting research question for further studies.

- preanalytical conditions are crucial for cfDNA: the authors should described which was the delay observed between blood sampling and freezing, and centrifugation conditions (one step ? two step ? temperature of centrifugation ? speed of centrifugation g ?)

**Reply:** We apologize for the confusion. As you have highlighted, indeed, sample preparation is critical to the quality of cfDNA. We have added more details from Line 217 to 223 as “…All blood samples were collected with Ethylenediaminetetraacetic acid (EDTA) tubes. After collection, plasma samples were stored at 4℃ and centrifuged at 4℃ within four hours. The blood samples were centrifuged at 1600g for 10 min at 4 °C, and the plasma was recentrifuged at 16,000g for 10 min at 4 °C. After centrifugation, plasma samples were immediately stored at −80 °C for further experiments. Samples of the lesion from the 14 surgically treated patients were also collected and stored at −80 °C.”

- at least, please indicate if all patients were included at diagnosis and systematically sampled before surgical and/or medical treatment and/or after beginning of treatment
**Reply:** Thanks for your kind reminder and sorry for the confusion. All patients were included at diagnosis, and all blood samples were collected before any surgical and/or medical treatment. We have added description to samples collection in section “2.2 Samples and processing” from Lines 209 to 210.

**Results**

This study gives interesting but very contrasting results between AE and CE patients, especially cfDNA yield and structure observed between the two species of *Echinococcus*. Except my previous limitations given previously, results were clearly expressed.

**Conclusions**

I disagree with author's statement (Lines 419-421): if puncture is prohibited for CE patients, it is of high diagnostic value and safe in AE patients. Please see
- Review in Clin Microbiol Review 2019 (Wen, Vuitton... your ref number one)
- Efficacy of ultrasound-guided core-needle biopsy in the diagnosis of hepatic alveolar echinococcosis: a retrospective analysis. Bulakci et al. Parasite 2016;23:19 (to be included in your references)
Include reference of crucial usefulness of imaging technique in diagnosis of echinococcosis
**Reply:** We apologize for the unclear expression in the manuscript and thank the reviewer for the kind suggestions. As you have pointed out, puncture is of high diagnostic value and safe in AE patients. What we wanted to highlight here is that “Traditional species identification of *Echinococcus* spp. in echinococcosis patients is always invasive, which relies on the product of surgery or puncture” from Line 542 to 543. However, while puncture is of high diagnostic value and safe in most AE patients, it is not recommended for CE patients, especially for CE4, CE5 and lung cysts, which may pose the risks of allergic reactions and anaphylaxis. We have modified the content and added the reference (Ref58) in the manuscript in Lines 543-548.

Imaging techniques are crucial for echinococcosis diagnosis in clinical, and we have added descriptions and references (Ref 7, Ref9, and Ref10) about this in Lines 131-133.

Last sentence of the paper is very interesting: "given the high sequencing depth and cost, the application scenarios and potential need to be further explored". Please develop what you mean.

**Reply:** We thank the reviewer for the suggestion. We have further added this part to the manuscript Lines 626-636.

Please balance more your results (even if very interesting contrast) : you only included one AE patient !! (Lines 411-412)

**Reply:** Thanks for your suggestion. We have revised the manuscript to emphasize the results from CE patients, and we have downplayed the description of the AE patient and made it more as a hypothesis to be tested in future studies.

**Editorial and Data Presentation Modifications**

Introduction: Please include some sentences about very young cysts, called CL (please refer to review of Wen, Vuitton and to Engler et al. Simple liver cysts and cystoid lesions in hepatic alveolar echinococcosis: a retrospective cohort study with Hounsfield analysis. Parasite 2019;26 :54)
**Reply:** Thanks for your suggestion, we have added a description of the Cystic Lesion (CL) from Lines 139 to 145.

Line 396 : Baraquin (instead of Baraquina)

**Reply:** We apologize for the spelling mistake, and we have corrected it in the manuscript.

**Summary and General Comments:**

As a summary, very interesting paper and well-done study. Authors should improve description of methods. If they processed blood from their AE patient with a distinct extraction, it is a bias. Thus, they should
- either clearly balance their results and discussion
- or limit their paper to their 22 CE patients (they may design another similar study focused on AE patients)

**Reply:** We thank the reviewer for the kind suggestions.

The description of the methods has been improved, especially the plasma centrifugation and cfDNA extraction. Please refer to the above reply, and the description was in Lines 217-222 and Lines 227-233.

The processing of the blood from the AE patient is exactly the same as the CE patients (as a matter of fact, he was diagnosed as CL stage with ultrasound). There was only one sample (N4) from the CE patients with larger volume plasma available, thus it was processed with another kit to extract cfDNA. As the AE blood sample was processed with the same kit as the other 21 CE blood samples, the special results of AE were not due to differences in the lab procedures or testing kits. The detailed information on plasma volume and kits were put in the S5 Table.

Since we just collected one AE sample by accident, we have downplayed the description and made it more as a hypothesis to be tested in futher studies. For the interesting phenomenon of the AE case, we hope our report can give some inspiration to other researchers with more AE samples.

**Concluding remarks:** Again, we thank the editor and reviewers for giving us the opportunity to strengthen our manuscript with your valuable comments and queries. We have worked hard to incorporate your feedback and hope that these revisions would have improved the quality of our manuscript.

Sincerely,

Jingkai Ji on behalf of all the authors ([jijingkai@genomics.cn](mailto:jijingkai@genomics.cn))

**References**

1.	Zhenghuan W, Xiaoming W, Xiaoqing L. Echinococcosis in China, a review of the epidemiology of *Echinococcus* spp. Ecohealth. 2008;5: 115–126. doi:10.1007/s10393-008-0174-0

2.	Parija S, Chaya D. Performance of polymerase chain reaction for the diagnosis of cystic echinococcosis using serum, urine, and cyst fluid samples. Trop Parasitol. 2014;4: 43. doi:10.4103/2229-5070.129164

3.	Baraquin A, Hervouet E, Richou C, Flori P, Peixoto P, Azizi A, et al. Circulating cell-free DNA in patients with alveolar echinococcosis. Mol Biochem Parasitol. 2018;222: 14–20. doi:10.1016/j.molbiopara.2018.04.004

4.	Moradi M, Meamar AR, Akhlaghi L, Roozbehani M, Razmjou E. Detection and genetic characterization of *Echinococcus* granulosus mitochondrial DNA in serum and formalin-fixed paraffin embedded cyst tissue samples of cystic echinococcosis patients. PLoS One. 2019;14: e0224501. doi:10.1371/journal.pone.0224501

5.	Weerakoon KG, McManus DP. Cell-Free DNA as a Diagnostic Tool for Human Parasitic Infections. Trends Parasitol. 2016;32: 378–391. doi:10.1016/j.pt.2016.01.006

6.	Tsai IJ, Zarowiecki M, Holroyd N, Garciarrubio A, Sanchez-Flores A, Brooks KL, et al. The genomes of four tapeworm species reveal adaptations to parasitism. Nature. 2013;496: 57–63. doi:10.1038/nature12031

7.	Kinkar L, Korhonen PK, Cai H, Gauci CG, Lightowlers MW, Saarma U, et al. Long-read sequencing reveals a 4.4 kb tandem repeat region in the mitogenome of *Echinococcus* granulosus (sensu stricto) genotype G1. Parasit Vectors. 2019;12: 238. doi:10.1186/s13071-019-3492-x

8.	Kinnings SL, Geis JA, Almasri E, Wang H, Guan X, Mccullough RM, et al. Factors affecting levels of circulating cell-free fetal DNA in maternal plasma and their implications for noninvasive prenatal testing. Prenat Diagn. 2015;35: 816–822. doi:10.1002/pd.4625

9.	Johnson JS, Spakowicz DJ, Hong BY, Petersen LM, Demkowicz P, Chen L, et al. Evaluation of 16S rRNA gene sequencing for species and strain-level microbiome analysis. Nat Commun. 2019;10: 1–11. doi:10.1038/s41467-019-13036-1
